# Stress-Induced Out-of-Context Activation of Memory

**DOI:** 10.1371/journal.pbio.1000570

**Published:** 2010-12-21

**Authors:** Karel Ježek, Benjamin B. Lee, Eduard Kelemen, Katharine M. McCarthy, Bruce S. McEwen, André A. Fenton

**Affiliations:** 1 Institute of Physiology, Academy of Sciences of the Czech Republic, Prague, Czech Republic; 2 Graduate Program in Neural and behavioral Science, State University of New York, Downstate Medical Center, Brooklyn, New York, United States of America; 3 Department of Physiology and Pharmacology, State University of New York, Downstate Medical Center, Brooklyn, New York, United States of America; 4 Rockefeller University, New York, New York, United States of America; 5 The Robert F. Furchgott Center for Neural and Behavioral Science, State University of New York, Downstate Medical Center, Brooklyn, New York, United States of America; 6 Center for Neural Science, New York University, New York, New York, United States of America; Mt. Sinai School of Medicine, United States of America

## Abstract

Inappropriate recollections and responses in stressful conditions are hallmarks of post-traumatic stress disorder and other anxiety and mood disorders, but how stress contributes to the disorders is unclear. Here we show that stress itself reactivates memories even if the memory is unrelated to the stressful experience. Forced-swim stress one day after learning enhanced memory recall. One-day post-learning amnestic treatments were ineffective unless administered soon after the swim, indicating that a stressful experience itself can reactivate unrelated consolidated memories. The swim also triggered inter-hemispheric transfer of a lateralized memory, confirming stress reactivates stable memories. These novel effects of stress on memory required the hippocampus although the memories themselves did not, indicating hippocampus-dependent modulation of extrahippocampal memories. These findings that a stressful experience itself can activate memory suggest the novel hypothesis that traumatic stress reactivates pre-trauma memories, linking them to memory for the trauma and pathological facilitation of post-traumatic recall.

## Abstract 2

An intensely stressful experience can itself activate memories that are unrelated to the stressful experience. This previously unknown property of stress could help explain how traumatic memories become pathological.

## Author Summary

This work identifies a powerful effect of stressful experiences on memories. We report that a single intensely stressful experience can activate memories in a situation that has essentially no physical or motivational relationship to the stressful experience. Using a forced-swim test as a stressor in rats, we find that this treatment was able to activate unrelated memories formed 24 hours earlier. We also find that the hippocampus of the brain is required for this effect of stress but that recall of the memories themselves does not. The ability of stress to activate memories that are unrelated to the stressful event may help to explain how memories can sometimes become pathological and uncontrollable following traumatic events, as in post-traumatic stress disorder. Our findings suggest the novel hypothesis that the stress of the traumatic event activates neutral, unrelated memories, which then become associated with the traumatic event. Subsequent normal recall of the neutral memories can more easily trigger inappropriate recall of the traumatic event, initiating another bout of stress and inappropriate associations of neutral and traumatic memories.

## Introduction

Inappropriate negative responses in emotionally neutral circumstances and the development of negative associations to harmless stimuli are core, debilitating features of post-traumatic stress disorder (PTSD), depression, and a host of anxiety and mood disorders. The possibility that stress itself might promote inappropriate associations between unrelated memories and events has not been explored, although a central role for stress and memory in these disorders is established [1]–[3]. Here we demonstrate that a single stressful experience can activate already consolidated memories outside of their appropriate context. These findings provide the basis for a novel hypothesis: by triggering out-of-context activation of memories stressful events themselves create opportunities for inappropriate associations to form, thereby promoting and perpetuating anxiety and mood disorders.

## Results

### Enhanced Memory after Stress

#### Experiment 1a

**Figure 1:**
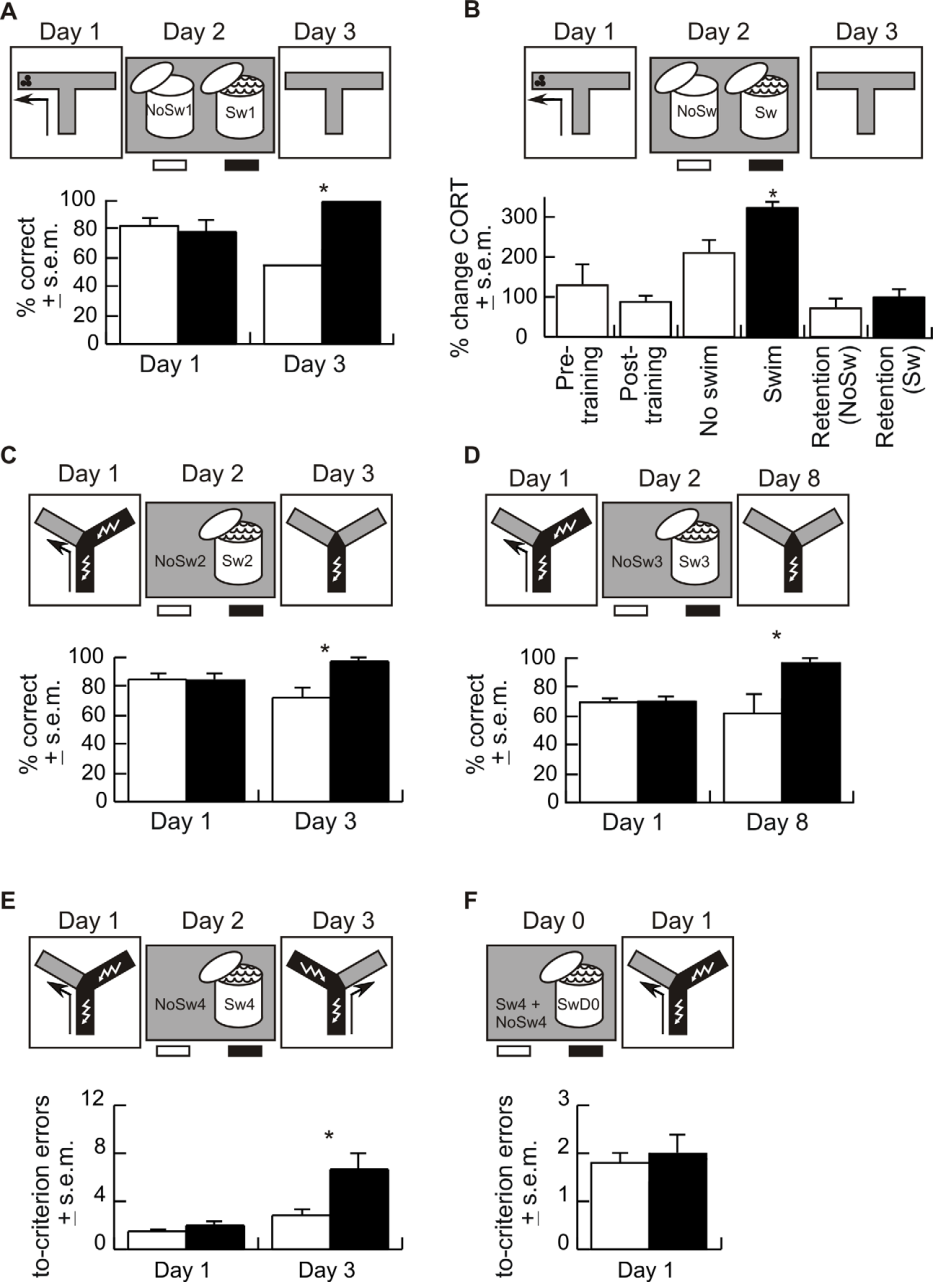
A stressful forced swim enhanced the expression of memory. (A) Experiment 1a—Rats trained on Day 1 in the appetitive left/right discrimination were either forced to swim (group Sw1, black, *n* = 8) or were put in the same bucket with only 1 cm deep water (group NoSw1, white, *n* = 9) on Day 2. Average percentage of correct responses in 5 acquisition trials and the percentage of rats that made a correct choice on the first extinction test trial are shown. Retention of memory, measured by the first choice, was better in the Sw1 group (χ^2^_1_ = 6.7; *p* = 0.01) (* *p*<0.01). (B) Experiment 1b—To measure circulating corticosterone levels, Experiment 1a was repeated and rats were sacrificed after Halothane anesthesia to collect trunk blood at different phases of the experiment, specifically before (*n* = 4) or after (*n* = 5) Day 1 training, after the Day 2 swim (*n* = 4) and no-swim (*n* = 5) procedures, and after the retention test on Day 3 in those that underwent swim (*n* = 7) or no swim (*n* = 6) on Day 2. A cage control group (*n* = 4) was removed from their cage and sacrificed. Individual corticosterone levels were normalized to that of the cage controls (229.8±47.5 ng/ml). The effect of experimental phase was significant (F_5,28_ = 19.8; *p* = 10^−8^). Dunnett post hoc tests comparing to pre-training levels only found corticosterone significantly elevated immediately after the swim (* *p* = 0.0001). (C) Experiment 2—One day after aversive left/right discrimination training in the short training protocol, the Sw2 group (black, *n* = 8) was forced to swim and the rats in the NoSw2 group (white, *n* = 10) were only handled. Five retention trials were given on Day 3. The average percent of correct responses on the acquisition and retrieval trials are plotted. The group that was forced to swim (Sw2) made more correct responses on Day 3 (t_16_ = 3.0, *p* = 0.008) (* *p*<0.01). (D) Experiment 2b—Experiment 2a was repeated this time, extending the interval between swim and the retention test to 6 d. The day after aversive left/right discrimination training in the short training protocol, the Sw3 group (black, *n* = 8) was forced to swim and the rats in the NoSw3 group (white, *n* = 10) were only handled. Five retention trials were given on Day 8. The average percent of correct responses on the acquisition and retrieval trials are shown. The group that was forced to swim made more correct responses on Day 8 than the group that did not swim (t_16_ = 2.95; * *p* = 0.009). (E) Experiment 3a—An aversive left/right discrimination memory was acquired during training on Day 1 using the intensive training protocol. On Day 2 the Sw4 group (black, *n* = 13) was forced to swim, and the NoSw4 controls were only handled (white, *n* = 11). Retention was tested on Day 3 by reversal training. The average number of to-criterion errors (4 consecutive correct trials—“4/4”) is plotted. On Day 3, the Sw4 group made more reversal errors (t_22_ = 2.4; *p* = 0.02), indicating an enhanced Day 1 memory (* *p*<0.05). (F) Experiment 3b—A new group of animals was forced to swim 24 h before initial Day 1 training (SwD0, *n* = 11). Learning was compared with the pooled Day 1 data of the Sw4 and NoSw4 rats (*n* = 24), which were only handled before training. The groups did not differ (t_33_ = 0.5; *p* = 0.6).

On Day 1, food-deprived rats were trained to perform left/right discriminations for food reward on a T-maze. On Day 2, one group of rats was forced to swim in a covered bucket for 20 min. The other group of rats was placed in the same bucket but with only 1 cm deep water so the animal was not forced to swim. On Day 3 all rats completed three non-reinforced T-maze trials to test retention of Day 1 memory. The groups did not differ on Day 1, (*p*>0.05), but retention measured as the first response on Day 3 was better in the group that was forced to swim (Figure 1A; *p* = 0.01). Although all the rats that were forced to swim chose the previously reinforced arm on the first trial, only 8 of their 16 choices on the second and third retention trials were to that arm (8/18 in the no-swim group). The rapid extinction of the conditioned response suggests that memory for the conditioned response was weak.

#### Experiment 1b

We investigated the possibility that the learning task itself is stressful, and therefore the stressful forced-swim experience may be serving as a memory activation cue. We repeated Experiment 1a, and at different stages of the protocol, we sacrificed animals to measure their corticosterone levels as an estimate of physiological stress. Relative to cage control levels, serum corticosterone increased over 300% immediately after the swim, which was the only significant increase of all the time points we assayed (Figure 1B; *p* = 0.0001). Stress, as estimated by corticosterone levels, was not significantly greater in animals prior to training than in cage controls. While the forced swim elevated corticosterone levels, the elevation did not persist to the next day at the time of the retention test, because corticosterone levels returned to baseline levels in animals that were forced to swim the day before. These data indicate that stress was uniquely high immediately after the swim, suggesting that the stress of the forced swim causes the enhancement of the left/right discrimination memory.

#### Summary

These results indicate the stressful forced swim enhanced the expression of the 24-h-old memory.

#### Experiment 2a

We next tested whether the swim would enhance memory for a negatively conditioned response using aversively reinforced left/right discrimination on a Y-maze. A “short” training protocol was used as follows: On Day 1, naïve rats were conditioned in four or five reinforced trials to make a left or right turn to avoid foot-shock (Figure 1C). On Day 2, one group of rats (Sw2) was forced to swim while control animals (NoSw2) were handled instead. In this and subsequent experiments, to reduce the opportunity for commonalities between the learning/recall and forced-swim experiences, the rats were not transported from the vivarium for the Day 2 treatments. Instead, on Day 2 the animals were given the forced-swim or control experience in the vivarium by a technician who did not participate in any other aspect of the study. The rat was removed from the home cage and placed in the bucket of water or a holding cage that was on the floor between 0.5 and 2 m below the location of the home cage. Thus, except for the forced swim itself and the handling associated with drying with paper towels that all animals received, this experience was not substantially different from the routine changing of the animal cages. On Day 3, all animals were given five unreinforced trials in the Y-maze to test for retention of the Day 1 memory. The groups did not differ on Day 1, but the Sw2 group scored more correct responses on Day 3 (Figure 1C; *p* = 0.008).

#### Experiment 2b

We repeated Experiment 2a, this time prolonging the interval between the forced swim and the retention test to 6 d in an effort to evaluate whether some lingering condition, like enhanced arousal at the time of the retention test, might account for the swim-induced enhancement of memory. Day 1 learning did not differ between the groups (Figure 1D), but Sw3, the group that was forced to swim, had significantly enhanced memory on Day 8 compared to NoSw3, the group that did not swim (t_16_ = 2.95; *p* = 0.009). The results were therefore similar whether retention was tested 1 or 6 d after the swim.

#### Summary

Once again, the forced swim enhanced the expression of the Day 1 memory. The enhancement did not depend on whether learning was appetitively (Expt. 1) or aversively (Expt. 2) reinforced. The memory enhancement was long lasting, at least for 6 d.

#### Experiment 3a

Experiment 3a tested whether the swim would enhance an already strong memory. To form a strong aversively reinforced left/right discrimination memory on Day 1, naïve rats received the “intensive” training protocol on the Y-maze as follows: They received training trials until they reached a criterion of 9 correct choices out of 10 consecutive trials and then they were given 30 additional trials. On Day 2 the rats were given either the forced swim (Sw4) or they were handled (NoSw4). On Day 3, the safe and punished arms were switched for reversal training, so the rat had to escape to the opposite arm than on Day 1. The reversal test was used to assess memory because after intensive training, control rats would perform perfectly on an extinction test, making it problematic to observe enhanced expression of memory. The groups did not differ on Day 1. The number of errors increased significantly from Day 1 to Day 3 in both groups (Sw4: t_12_ = 3.5, *p* = 0.004; NoSw4: t_10_ = 3.3, *p* = 0.008), which was expected on the assumption that the memory acquired on Day 1 would interfere with learning the conflicting response on Day 3. Importantly, the swim on Day 2 caused a larger increase in the number of errors on Day 3 compared to the group that did not swim (Figure 1E; *p* = 0.02), consistent with the possibility that the swim had enhanced the Day 1 memory.

#### Experiment 3b

In Experiment 3a, either the stressful swim enhanced the expression of left/right discrimination memory as it did in Experiments 1 and 2, or the swim impaired left/right discrimination learning on the reversal test. Experiment 3b was performed to distinguish between these possibilities. Rats were forced to swim and then 24 h later given intensive left/right discrimination training (SwD0). Learning in this group was not different from the initial learning of the groups in Experiment 3a (Sw4 and NoSw4; *p*>0.05; Figure 1F), showing that the swim stress neither impaired nor enhanced the ability to learn the discrimination task. These results indicate the swim in Experiment 3a enhanced memory rather than impaired learning.

#### Summary

Experiments 1, 2, and 3 all demonstrate the forced swim enhanced the expression of memory. This phenomenon was robust; the memory enhancement persisted at least 6 d. It was observed for both appetitive and aversive conditioning, for weak and strong memories, and whether memory was assessed by extinction or reversal tests.

### Stress Activates Consolidated Memories

Stress modulates memories that are undergoing cellular consolidation [4] and may play a role in the swim-induced enhancement of memory. Experiments 4 and 5 were designed to test if the day-old memory is undergoing cellular consolidation at the time of swim. If the memories were consolidating, then amnesic treatments by electro-convulsive shock (ECS) [5] or the beta-adrenergic antagonist Propranolol [6] 24 h after conditioning should impair retention.

#### Experiment 4

On Day 1, the intensive training protocol was used to condition an aversively reinforced left/right discrimination in naïve rats. On Day 2, the rats were divided into five groups. To replicate the enhancing effect of swim (Experiment 3a), one group of rats was only handled (NoSw-NoECS) and a second group was forced to swim (Sw-NoECS). To test if the memory was consolidating 24 h after training, rats in a third group were given ECS and not forced to swim (NoSw-ECS). To examine the effect of ECS after the swim, rats in the remaining two groups were given ECS immediately (Sw-ECS) or 5 h after swim (Sw-delECS).

**Figure 2:**
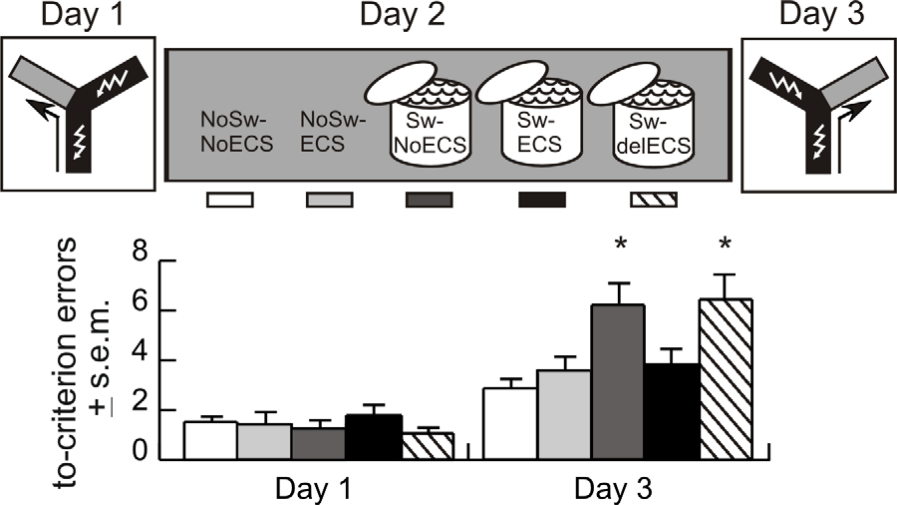
Experiment 4—Electroconvulsive shock blocked the swim-induced enhancement of memory. All rats were trained in the intensive training protocol on Day 1. On Day 2, ECS was delivered immediately after the swim in the Sw-ECS group (black, *n* = 16), 5 h after the swim in the Sw-del-ECS group (diagonal stripes, *n* = 13) or without the forced swim experience in the NoSw-ECS group (light gray, *n* = 11). Rats in the NoSw-NoECS group were only handled (white, *n* = 11) and animals from the Sw-NoECS group (medium gray, *n* = 12) only swam. On Day 3, the rats were tested in the reversal paradigm. The average number of to-criterion (4/4) errors on Days 1 and 3 are plotted. The groups did not differ on Day 1, but they differed on Day 3 (F_4,58_ = 4.5, *p* = 0.003). The Sw-NoECS and Sw-delECS animals expressed stronger memory during the reversal test in comparison to all other groups (all *p*s <0.05). Performance of the NoSw-ECS, Sw-ECS, and the NoSw-NoECS groups was not different (*p*>0.05) (* *p*<0.05).

On Day 1, the groups did not differ. Day 3 testing replicated the significant group effect of Experiment 3a (F_4,58_ = 4.5, *p* = 0.003). The swim enhanced the expression of memory because the Sw-NoECS group was different from the groups that did not swim (post hoc, *p*s <0.05; Figure 2).

The ECS in the NoSw-ECS group did not alter the expression of memory because their performance was not different from the NoSw-NoECS group's performance (post hoc, *p*>0.05). This indicated the memory was not labile and thus not consolidating 24 h after conditioning.

Unexpectedly, the Sw-ECS group differed from the Sw-NoECS and Sw-delECS groups (post hoc, *p*s <0.05), indicating that soon after the swim, memory was sensitive to ECS. ECS reduced performance in the Sw-ECS group to the level of the rats that did not swim (post hoc, *p*s >0.05). The effect of ECS was not observed 5 h after the swim because performance in the Sw-NoECS and Sw-delECS groups did not differ (post hoc, *p*>0.05).

#### Summary

The results together suggest that the swim activated a stable memory, making it transiently sensitive to amnestic treatment. However, it is also possible that the rapid reversal learning caused by forced swim followed immediately by ECS is a result of a change such as increased arousal that persisted at the time of the retention test.

#### Experiment 5

Experiment 5 was designed to test the hypothesis suggested by the results of Experiment 4, that the swim makes an already consolidated memory sensitive to amnestic treatment. Propranolol reliably causes amnesia for a recently activated one-trial inhibitory avoidance memory [6]. If the stressful swim activates consolidated memory, then Propranolol should not affect retention of inhibitory avoidance in rats that did not swim, but it should cause amnesia if it is administered soon after the swim is administered.

Inhibitory avoidance was tested using a box with separate bright and dark compartments. On Day 1, naive rats were conditioned by foot-shock to inhibit the preference for entering the dark compartment. On Day 2, the rats were divided into five groups. To test whether the memory already consolidated within 24 h, the first group was injected (1 ml/kg i.p.) with saline (NoSw-Sal) and the second group was injected with Propranolol (NoSw-Pro). To test whether the swim made the memory sensitive to Propranolol, the third group was forced to swim and then injected with saline (Sw-Sal), and the fourth and fifth groups were forced to swim and then injected with Propranolol either immediately (Sw-Pro) or 5 h (Sw-delPro) after swim. The Propranolol dose administered was 10 mg/ml/kg i.p. [6]. Conditioned inhibitory avoidance was tested in all groups without reinforcement on Day 3.

**Figure 3:**
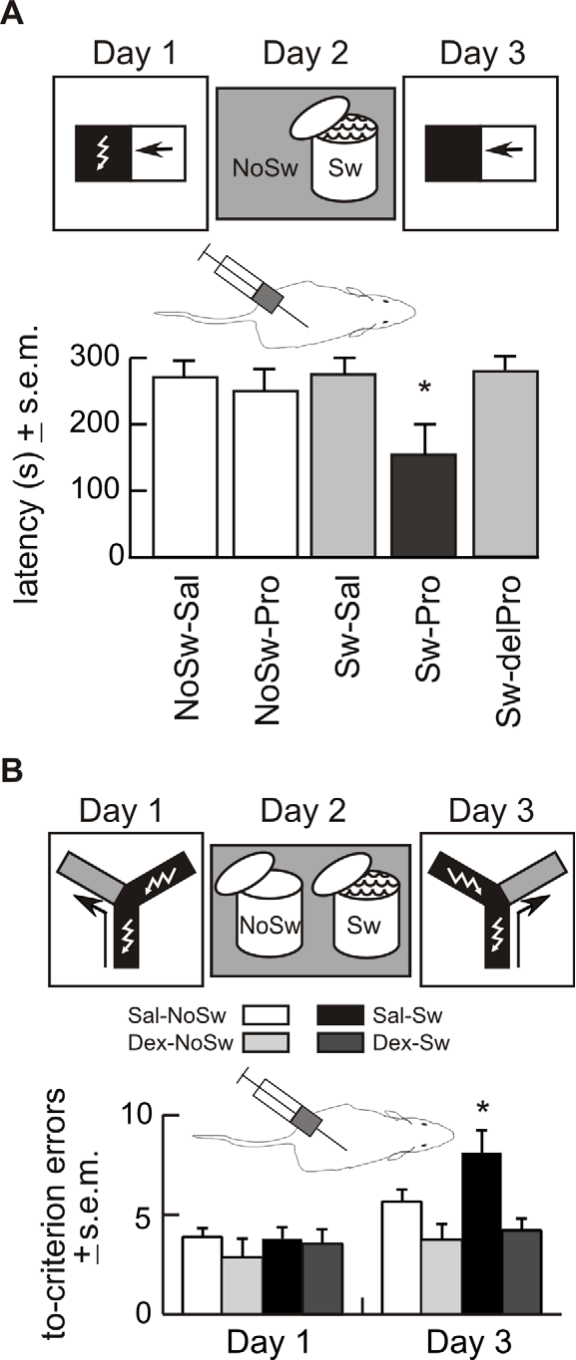
Experiments 5 and 6—Stress is necessary for the forced swim to alter consolidated memories. (A) Experiment 5—Propranolol caused amnesia of inhibitory avoidance memory only if it was administered after the forced swim. Rats were trained in the inhibitory avoidance paradigm on Day 1. On Day 2, they were either forced to swim (Sw) or just handled (NoSw), and immediately afterwards injected with 10 mg/ml/kg Propranolol or saline (NoSw-Sal *n* = 11, NoSw-Pro *n* = 10, Sw-Sal *n* = 10, Sw-Pro *n* = 11). Rats in the Sw-delPro group (*n* = 12) were injected with Propranolol 5 h after the swim. The average ± step-through latencies on Day 1 were: NoSw-Sal = 8.5±1.7; NoSw-Pro = 6.5±0.79; Sw-Sal = 7.4±1.5; Sw-Pro = 5.5±0.91; Sw-delPro = 7.9±0.98). The step-through latencies recorded on Day 3 are plotted. The groups did not differ on Day 1, but they differed on Day 3 (F_4,49_ = 3.0; *p* = 0.03). Amnesia, manifested as reduced step-through latencies, was observed only in the Sw-Pro group (all post hoc *p*s <0.05) (* *p*<0.05). The data indicate the swim activated the consolidated memory. Whether or not inhibitory avoidance was enhanced by the swim could not be determined in this experiment because performance was already maximal after the single conditioning trial. The data cannot be explained by previous work showing that exposure to a novel alerting stimulus can enhance retrieval of conditioned inhibitory avoidance because in that case, beta-endorphin activation triggered beta-noradrenergic and cholinergic processes that acted at the time of retrieval only if the retrieval test was given within less than 6 h [63],[64]. (B) Experiment 6—Dexamethasone blocks the swim-induced enhancement of memory. Rats were trained in the intensive left/right discrimination on Day 1. The next day, Dexamethasone (Dex; 0.2 mg/kg i.p.) or saline (Sal) was administered 2 h prior to the forced swim (Sw) or no swim (NoSw) shallow-water control treatments. Retention of the Day 1 left/right discrimination memory was tested on Day 3 by the reversal test. Enhanced memory was observed in the saline-treated animals that were forced to swim (Sal-Sw), but the effect was blocked by the action of Dexamethasone in the (Dex-Sw) group. (* *p*<0.05 compared to the saline-treated no swim (Sal-NoSw) control group).

On Day 1, all animals rapidly moved to the dark compartment and step-through latencies were not different between the groups (all averages <10 s; see Figure 3 legend for details). On Day 3, the rats avoided entering the dark compartment as the step-through latencies were prolonged to over 100 s in all groups. The paired *t* tests comparing the day 1 and 3 latencies of each group were all significant, ranging from *t* = 4.2, *p* = 0.0002 for the Sw-Pro group to *t* = 27.4, *p* = 10^−11^ in the Sw-delPro group. Importantly, avoidance differed between the groups (F_4,49_ = 3.0, *p* = 0.03) because the latency was lower in the Sw-Pro group (Figure 3). Importantly, avoidance was not different in the NoSw-Sal and NoSw-Pro groups (post hoc, *p*>0.05). This indicated the memory had consolidated 24 h after training and that Propranolol administration itself did not interfere with the expression of the conditioned response. Avoidance was attenuated in the Sw-Pro rats compared to all the other groups (post hoc, *p*s <0.05). This indicated that Propranolol caused amnesia if it was injected immediately but not 5 h after the swim (Figure 3A).

Propranolol attenuated inhibitory avoidance to a level that was below the level of the no swim control rats, suggesting the original memory was disrupted. In contrast, ECS administration in Experiment 4 attenuated the enhanced left/right discrimination to the level of the no swim controls, suggesting ECS disrupted the updating but not the original avoidance memory. These differences may be attributed to the treatment doses, the task differences in left/right discrimination, and inhibitory avoidance and/or differences in the brain regions that are critical for these behaviors. Left/right discrimination, for example, is not sensitive to hippocampal dysfunction (see Experiment 8), while inhibitory avoidance is [7]. Despite differences in Experiments 4 and 5, both results converge on the fact that a 24 h latent conditioned response was insensitive to amnestic treatment before, but not after, the swim.

#### Experiment 6

Propranolol blocks the adrenergic component of stress, so the effect of the drug on memory in Experiment 5 also suggests that stress can alter a stable, consolidated memory. We used Dexamethasone, a potent suppressant of the hypothalamic-pituitary-adrenal (HPA) axis to investigate further whether the stress of the forced swim triggers the memory alteration (Figure 3B). On Day 1, rats (*n* = 36) received left/right discrimination training in the intensive aversive protocol. On Day 2, half the rats were injected with Dexamethasone (0.2 mg/ml/kg) and the other half with saline. Two hours later, half the rats treated with Dexamethasone and half those treated with saline were forced to swim for 20 min. The remaining rats were put in the bucket with shallow water. Retention of the Day 1 left/right discrimination memory was tested on Day 3 using the reversal test. Dexamethasone blocked the enhancement of memory that was observed in the saline animals that swam. There was a significant interaction between day and group (F_3,64_ = 3.15, *p* = 0.03). Post hoc tests confirmed that the saline-treated rats that swam showed increased retention, but the Dexamethasone-treated rats did not. These data provide additional evidence that stress itself involving both catecholamines and adrenal steroids is necessary for the memory-enhancing effect of the forced swim.

#### Summary

Using different memory paradigms, Experiments 4–6 revealed that the swim made conditioned avoidance susceptible to amnestic treatment, and activation of both the adrenergic and HPA components of stress are crucial for the phenomenon. The failure of ECS and Propranolol to affect the day-old memory in Experiments 4 and 5 caused us to reject the hypothesis that memory was undergoing cellular consolidation at the time of the forced swim. Nonetheless, the forced swim improved expression of left/right discrimination memory and made the expression of conditioned avoidance memories sensitive to ECS and Propranolol, phenomena that are normally triggered by memory activation [5],[8].

Together, Experiments 1–6 demonstrate that a stressful swim reactivates consolidated memories causing them to be strengthened or, alternatively, causing them to weaken when an amnestic treatment followed the swim. While we are not certain of the mechanism, these results can be explained if the swim-elicited stress response coupled with the activated memory to enhance reconsolidation [9],[10] and strengthen the memory. This possibility would account for why such an out-of-context activation of memory has not been reported by others. Out-of-context memory activation would go unnoticed unless it was activated in conditions that promoted its enhancement or disturbance. As demonstrated in Figure 1B, the forced swim is stressful [11], which can reinforce synaptic plasticity by transforming early-LTP to late-LTP [12]. Post-learning stress improves consolidation and subsequent retrieval of memory [4], so the presence of circulating stress hormones at the time of swim-induced activation of memory would be expected to enhance consolidation and therefore retrieval. According to this interpretation, the memory enhancement is secondary to the activation of memory. We used the inter-hemispheric transfer (IHT) experimental paradigm to seek evidence that the swim activated memory independently of a memory enhancement.

The hypothesis that discrimination memory was activated by the forced swim was tested using the phenomenon of IHT of lateralized memory. Learning under functional hemidecortication when one (“non-trained”) hemicortex is inactivated causes a “lateralized memory state,” in which subsequent expression of the memory relies on the (“trained”) hemicortex that was active during learning [13]–[15]. When tested with the trained hemicortex inactivated, the subject behaves as if naïve. Lateralized memories cease to be lateralized if the memory is activated by returning the subject to the learning context with both hemispheres functioning [13],[16],[17]. Afterwards, the once lateralized memory can be expressed independently of the hemisphere that was active during learning. This phenomenon is called IHT of lateralized memory. Since memory activation is necessary to induce IHT of a lateralized memory [17], we used IHT as an assay for whether the swim activated left/right discrimination memory.

#### Experiment 7

The hypothesis that the swim activates memory makes two strong predictions. First, if the forced swim activates memory, then it should induce IHT. The second prediction is that IHT should be blocked by inactivating the trained hemisphere to prevent memory activation during the swim.

On Day 1, left/right discrimination memory was lateralized using the protocol of Goldowitz et al. [18]. Naïve rats were given intensive left/right discrimination training in the Y-maze under unilateral cortical spreading depression (CSD) [19]. On Day 2, the animals were divided into three groups. One group (Lat-Sw) was forced to swim to activate memory according to the hypothesis. Another group was also forced to swim, but CSD was induced in the trained hemispheres (opposite side as on Day 1) to prevent memory from activating during the swim (Lat-Sw-CSD). The third group was only handled (Lat-NoSw). According to the hypothesis, memory would not be activated in this group and so IHT would not occur. Two hours after the swim, CSD was elicited in the trained hemisphere of all animals and they were tested for IHT by reversal training.

**Figure 4:**
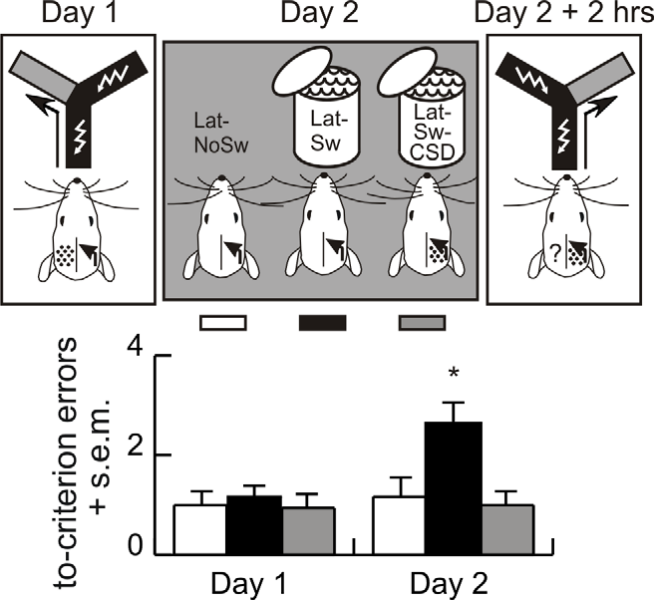
Experiment 7—Swim-induced interhemispheric transfer of lateralized memory. The intensive training protocol was administered under unilateral CSD (shading) on Day 1, which led to the formation of a lateralized left/right discrimination memory (arrow). The next day the forced swim was administered with an intact brain in group Lat-Sw (black, *n* = 25) and under CSD in the opposite hemisphere as during training in group Lat-Sw-CSD (gray, *n* = 13). The rats of Lat-NoSw group (white, *n* = 11) were only handled. Two hours later, reversal training was used to test the expression of memory with the originally trained hemisphere inactivated by CSD and the untrained side (question mark) functional. The average numbers of to-criterion (4/4) errors on Days 1 and 2 are presented. There were significant effects of group (F_2,46_ = 5.9; *p* = 0.005), day (F_1,46_ = 4.1; *p* = 0.05), and the interaction (F_2,46_ = 3.2; *p* = 0.05). On Day 1 the groups did not differ. On Day 3 the error scores were greater in the Lat-Sw group compared to the Lat-CSD-Sw and Lat-NoSw groups (F_2,46_ = 6.5; *p* = 0.003; post hoc, both *p*s <0.05), which were similar (* *p*<0.05). The data indicate the 1-d-old memory was activated by the swim.

The groups did not differ on Day 1 (Figure 4). On Day 2, the groups differed (F_2,46_ = 6.5, *p* = 0.003) because the Lat-Sw group made significantly more errors compared to the Lat-Sw-CSD and Lat-NoSw groups. The number of errors increased from Day 1 to Day 2 only in the Lat-Sw group (t_24_ = 3.5, *p* = 0.002), indicating that IHT only occurred in this group. Memory remained lateralized in the Lat-NoSw and Lat-Sw-CSD groups because Day 1 training did not influence behavior on Day 2. The observation of IHT in the Lat-Sw group but not in the Lat-NoSw group (t_10_ = 0.4, *p* = 0.7) indicates the swim triggered memory activation and the memory was active during the swim. CSD elicited in the trained side prevented memory activation and this blocked the swim-induced IHT in the Lat-Sw-CSD animals. These results confirm the hypothesis that memory was activated during the swim.

#### Summary

The swim modified discrimination memory by enhancing its expression, by switching it from a consolidated to a labile state, and by modifying what part of the brain could retrieve it, a process thought to require synapse-specific plasticity. We conclude that the stressful swim activated memory.

In principle, rodent memory activation could be triggered by internal variables like the level of a circulating hormone [20] or by complex internal and subjective variables that are invisible to objective observers. This makes the swim-induced activation of memory all the more remarkable because the triggering experience did not need to have any physical contextual elements in common with the experience of the memory encoding or retrieval. Accordingly we called the swim-induced activation of memory “out-of-context activation of memory” (OCAM). At least on the surface, OCAM is a common feature of human episodic recall, which is typically triggered by any number of internal variables including introspection. The episodic encoding and recall associated with human conscious recollection is impaired by hippocampal dysfunction [21]–[23]. While our results provide no insight into the subjective experience of OCAM, they provide a definitive test of whether the hippocampus is necessary for OCAM.

### OCAM Requires Hippocampus

Temporary inactivation of hippocampus with long-acting (6–10 h) tetrodotoxin (TTX) [24] or short-acting (∼30 min) lidocaine [25] was used to test if hippocampus is important for OCAM. The TTX injection was determined to block neural activity in both the dorsal and the ventral hippocampi [24].

#### Experiment 8a

**Figure 5:**
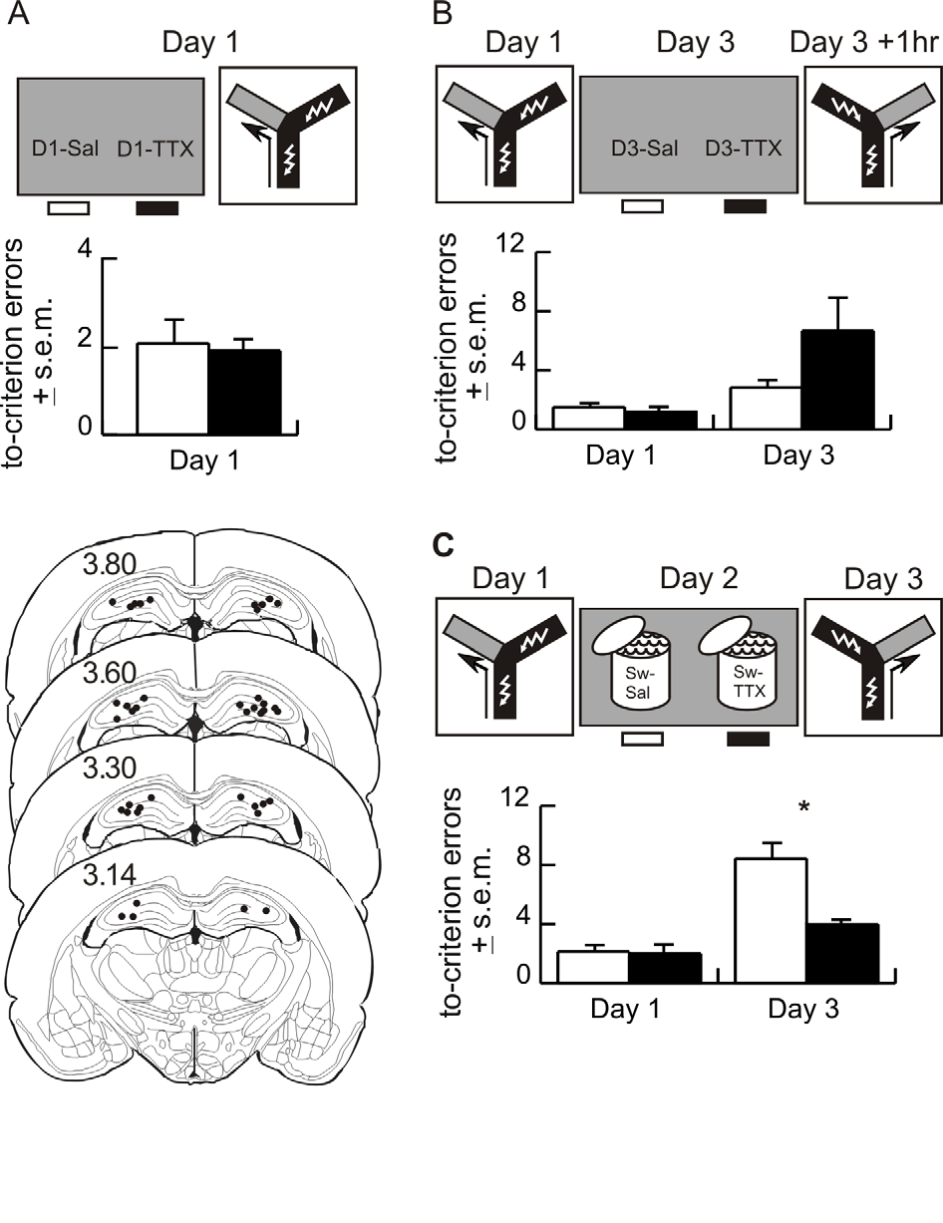
Experiments 8 and 9—Acquisition and retrieval of left-right discrimination does not depend on hippocampus but the swim-induced enhancement of memory does. (A) Experiment 8a—Bilateral TTX inactivation of dorsal hippocampus in the D1-TTX (black, *n* = 9) group did not influence left/right discrimination learning in the Y-maze task compared with saline controls (D1-Sal, white, *n* = 11; *p*>0.05). (B) Experiment 8b—Another two groups of animals were trained on Day 1 with the intensive training protocol. One hour before the Day 3 reversal test, TTX (D3-TTX, black, *n* = 7) or saline (D3-Sal, white, *n* = 11) was infused into both dorsal hippocampi. The TTX injection did not impair retrieval. In fact, there was an opposite tendency for enhanced retrieval in the hippocampus-inactivated group [65], but the trend did not reach significance (*p*>0.05). Thus hippocampus was not necessary for learning or expressing left/right discrimination memory. (C) Experiment 9—Hippocampus was necessary for the swim-induced enhancement of memory. Left/right discrimination was conditioned on Day 1 using the intensive training protocol. On Day 2, rats received bilateral intrahippocampal injections of saline (Sw-Sal, white, *n* = 11) or TTX (Sw-TTX, black, *n* = 8), and 1 h later they were forced to swim. Memory was tested by reversal training on Day 3. The numbers of to-criterion errors are reported. The TTX injection attenuated the swim-induced memory enhancement (t_17_ = 3.47; *p* = 0.003) (* *p*<0.01). The placement of 20 bilateral injections are depicted on schematic coronal sections [66]. The number indicates the section's location posterior to bregma.

The intensive training protocol was used to first determine whether bilateral hippocampal inactivation by TTX impairs Day 1 learning in naïve rats. Injecting TTX (D1-TTX, *n* = 9) 1 h before intensive left/right discrimination training did not alter Day 1 learning compared to saline-injected (D1-Sal, *n* = 11) controls (Figure 5A).

#### Experiment 8b

The intensive training protocol and reversal test were next used to determine whether bilateral hippocampal inactivation by TTX impairs Day 3 retrieval. Eighteen naïve rats were given intensive left/right discrimination training on Day 1. On Day 3, an hour before reversal training, rats were injected in both hippocampi either with TTX (D3-TTX) or saline (D3-Sal). The groups were not different on Day 1. The TTX injection did not impair Day 3 retrieval compared to the saline-injected controls (t_16_ = 1.8, *p* = 0.09; Figure 5B).

#### Summary

Experiments 8a and 8b demonstrated that the left/right discrimination memory could be acquired and recalled independently of the hippocampus. This put us in a position to ask whether hippocampus is necessary for OCAM itself.

#### Experiment 9

If the hippocampus is important for OCAM, then bilateral inactivation of hippocampus during the stressful swim should block the swim-induced enhancement of memory. Naïve rats received intensive left/right discrimination training on Day 1. On Day 2, they received bilateral injections of either TTX (Sw-TTX) or saline (Sw-Sal) in the dorsal hippocampi. One hour later, all rats were forced to swim for 20 min. On Day 1, the groups did not differ. On the Day 3 reversal test (Figure 5C), the Sw-TTX group had significantly weaker memory than the Sw-Sal control group (t_17_ = 3.5; *p* = 0.003).

#### Summary

These data suggest the hippocampus was necessary for the swim-induced memory enhancement.

#### Experiment 10

**Figure 6:**
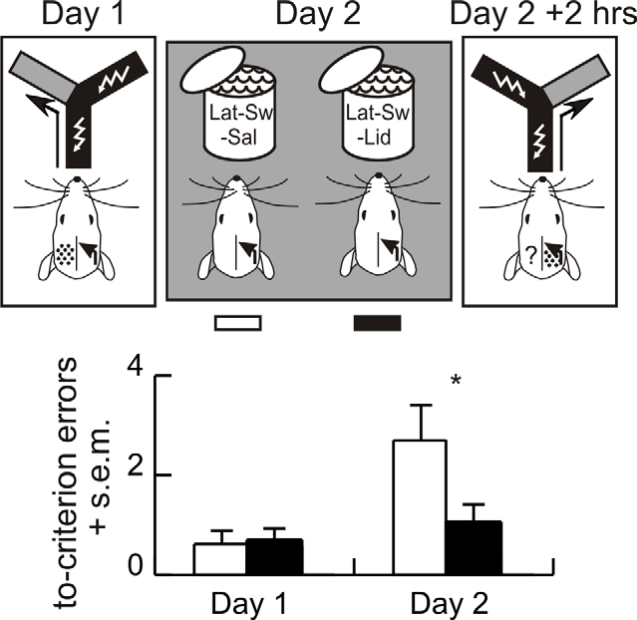
Experiment 10—The swim-induced inter-hemispheric transfer of lateralized memory required hippocampal function. Left/right discrimination was conditioned on Day 1 using the intensive training protocol with one hemicortex inactivated by cortical spreading depression (CSD). On Day 2 rats received bilateral hippocampal injections of saline (Lat-Sw-Sal, white, *n* = 11) or lidocaine (Lat-Sw-Lid, black, *n* = 21), and then they were forced to swim. Memory was assessed by reversal training 2 h after the swim with the originally trained hemicortex inactivated by CSD. The number of to-criterion errors is reported. The groups did not differ on Day 1 but they differed on Day 2 (t_30_ = 2.27; *p* = 0.03). The Day 1 memory was lateralized because rats in the Lat-Sw-Lid group performed as if naïve on Day 2. The swim induced IHT of the lateralized memory because rats in the Lat-Sw-Sal group made more reversal errors on Day 2 (* *p*<0.05).

If the hippocampus is necessary for OCAM, then bilateral inactivation of hippocampus during the swim should also block swim-induced IHT of a lateralized memory. On Day 1, naïve rats received intensive left/right discrimination training under CSD in one hemicortex to cause lateralized memory formation. On Day 2, 21 rats received bilateral intrahippocampal injections of lidocaine (Lat-Sw-Lid) and 11 rats were injected with saline (Lat-Sw-Sal). Lidocaine was used instead of TTX because lidocaine only blocks neural transmission for ∼30 min. Immediately after the injection, the rats were forced to swim. Two hours later CSD was elicited in the opposite side to the Day 1 training and memory was assessed by the reversal test. The groups did not differ on Day 1; the groups did differ on Day 2 (t_30_ = 2.3, *p* = 0.03). Only the saline-injected group demonstrated IHT by expressing Day 1 memory (t_10_ = 3.0, *p* = 0.01; Figure 6).

#### Summary

Because the lidocaine-injected rats expressed no Day 1 memory, this result indicates that OCAM required a functional hippocampus during the swim.

## Discussion

### OCAM

The results of this set of experiments suggest that stress can activate memory, even if the memory is unrelated to the stressful experience. We use the term “memory activation” in the established sense that the term is used in the consolidation and reconsolidation literatures, to mean that memory is in a labile (“active”) state rather than an inert (“inactive”) state [9]. We provided multiple lines of evidence that a stressful swim returned a consolidated memory to a labile state. As a result, expression of the memory was strengthened, and if the memory was lateralized, the swim triggered its interhemispheric transfer.

The activation by forced-swim stress was independent of the conditioned and external contextual stimuli that were present during learning, leading us to call the phenomenon OCAM (alternative interpretations are considered and rejected in the section that follows). OCAM seems to be a general phenomenon that does not depend on whether the conditioned response is rapidly extinguished (Experiment 1) or persistent (Experiments 2–10) or whether the activated memory is acquired during single (Experiment 5) or multiple (all other experiments) appetitively (Experiment 1) or aversively (all other experiments) conditioned trials that reinforce an inhibitory (Experiment 5) or an active (all other experiments) conditioned response. OCAM also seems to occur whether or not memory expression depends on the hippocampus (inhibitory avoidance) or the neocortex (left/right discrimination). Both beta-adrenergic activation (Figure 3A) and Dexamethasone-suppressible HPA activity (Figure 3B) were required for the OCAM effect, indicating a central role for stress and two key stress mediators. This is consistent with the idea that stress and arousal act together to modulate memory mechanisms [26]–[28].

### Alternative Interpretations

#### Arousal

Forced swimming is probably arousing, in which case, can the results be explained by a post-learning facilitation of memory caused by enhanced arousal during the retrieval test 1 to 6 d after the swim? We think this explanation is unlikely for several reasons. To our knowledge, the longest reported post-training interval for an effective memory facilitating treatment is 6 h, and no treatments have been effective 9 h after learning [29]. The effective limit of several hours is probably because such treatments affect the time-limited molecular mechanisms that stabilize memory. The pre-training forced swim did not affect learning the next day, which does not support the idea that arousal was enhanced a day later, at the time of the retention test (Figure 1E). Nonetheless, one might argue that it is still possible that arousal due to the post-training swim is exceptional because the swim was facilitating from 24 h to 6 d (Experiment 2b) after learning. However, without assuming that arousal activates consolidated memory, arousal does not account for why 24 h post-training ECS (Experiment 4) prevented memory facilitation if applied immediately after but not 5 h after the swim. Neither does arousal itself account for why a beta-adrenergic antagonist (Experiment 5) was only amnestic soon after the forced swim. Despite its non-mnemonic central, cardiac, and autonomic effects, Propranolol given without swimming or long after swimming did not affect memory retrieval. One might postulate that both Propranolol and ECS could have altered arousal on the retention test a day later, but neither treatment affected memory expression if they were not administered soon after the forced swim, indicating altered arousal is not sufficient to explain the OCAM effect. In contrast, both treatments affected memory expression when administered soon after the swim, suggesting the swim made the memory labile and vulnerable to the treatments. It is even harder to explain how arousal itself triggered the IHT phenomenon, which changes the localization of a memory, not merely its strength (Experiment 7).

#### Persistent hormonal changes alter retention or retrieval without activation

The corticosterone data as well as the ineffectiveness of Propranolol administration 5 h after the swim (Experiment 5) make it unlikely that persistently altered stress hormone changes alone account for the swim-induced memory activation and enhancement. Although unlikely, it is nonetheless still possible that other endogenous hormonal changes persisted after the swim and account for the enhanced memory on the retention tests 24 h or even 6 d later (Experiment 2b), for example by inducing preservative behavior. All the results, however, cannot be readily explained by some unidentified change in hormonal expression at the time of the retention tests. It is particularly difficult for the possibility of a persistent hormonal change to explain the IHT results of Experiments 7 and 10 because the swim triggered IHT, which is not merely an enhancement of memory or retrieval but a change in the information content that can be localized to a brain region.

#### Stress or other persistent hormonal changes as a reminder cue

It is unlikely that stress itself could have served as a reminder cue for activating the conditioned response. At least in Experiment 1, endogenous corticosterone levels were distinct between the learning and swim experiences. Levels were elevated by 300% by the forced swim, but compared to the levels of naïve animals taken from the cage and sacrificed, corticosterone levels were unaltered by either appetitive learning or retrieval regardless of the intervening swim experience. While it is still possible that an unidentified stress-triggered hormonal change acted as a reminder for the memory activation, this hypothesis is not falsifiable. We formulated the alternative, falsifiable hypothesis that memory was activated out-of-context. Even within the literature on endogenous state-dependent learning and recall, we are unaware of any other reports of memory activation in the absence of the external stimuli that were present during learning. If the OCAM hypothesis is falsified, it will be valuable to learn what stimulus was the reminder that triggered memory activation during the swim. At the very least, this may provide an experimental model of the mental process that in people appears to be context-free recall.

#### Reminder cues present during the forced swim

Although some features of the forced-swim procedure were common to the training and retention procedures such as handling by a human, the results of the control animals contradict the possibility that uncontrolled external features cued the swim-induced memory activation. The behavioral testing and swim environments were different rooms to minimize contextual similarities. Although in the first experiments, all the animals were transported from the vivarium for both the training and the swim procedures, the rats that received the control swim procedures did not show evidence of memory activation. In subsequent experiments, we excluded the possibility that transport from the vivarium to the laboratory was the trigger for the memory activation by putting the rats to swim in a bucket that was placed just below their home cage in the vivarium. The swim still elicited a robust enhancement of memory in all these experiments. Furthermore, evidence of memory activation was not observed in any of the many control groups, even though the control animals received the same environmental exposures and handling as the rats that were forced to swim, with the exception of swimming. These procedures included transport the rare times it was done, being manipulated by hand, and actions to dry the fur with paper towels. It is nonetheless possible that some feature of being in the bucket with deep water was not reproduced by the control swim procedure of being in a similar bucket with shallow water, and that feature was common to the L/R discrimination or inhibitory avoidance learning and sufficient to activate the relevant memory.

#### No parsimonious alternative to OCAM and its possible biological utility

We see no alternative to concluding that the forced swim activated a consolidated memory in the absence of external conditioned and contextual stimuli and in the absence of the conditioned response. Neither external conditioned nor contextual stimuli were present during the swim to elicit the conditioned responses, and the rat could not express these responses during the swim. While the rule of parsimony requires concluding that OCAM occurs in rats, this conclusion may be unintuitive. It seems, however, that stress-triggered OCAM could provide the basis for an obviously adaptive biological advantage. People commonly review recent past experience in response to current adversity in the effort to identify the cause of the adversity and increase the possibility of avoiding it in the future. This cognitive ability would also confer an adaptive advantage to lower animals. This ability would benefit from causal reasoning, and there is evidence, albeit inconclusive, that causal reasoning occurs in rats [30]. However, it is also possible that merely by activating recent memories in response to a life-threatening experience, an organism can improve its chances to avoid the danger in the future should it escape. This adaptive ability would occur because increased levels of circulating stress hormones will act to enhance the activated set of associations that led to the aversive, threatening situation and the effective escape response. According to this speculation, OCAM could provide for this ability without the need for causal reasoning or conscious recollection. While there are other possibilities for why forced swimming might trigger OCAM, accepting the conclusion that this occurs must be based on reproducible experimental observations and their parsimonious interpretation rather than on speculations about why OCAM may or may not occur.

### Does OCAM Affect Memory Storage or Retrieval?

The forced swim modified a consolidated memory and no anterograde learning effects were detected (Experiment 3b). The results of the first three experiments that assayed enhanced memory expression did not distinguish between whether the forced swim had its effect on memory storage or the process of memory retrieval, including for example by inducing perseveration during the reversal tests for memory strength. However, the results of Experiments 4 and 5 with amnestic agents indicate the swim-induced modifications only occurred soon after the swim but not after a 5-h delay. This strongly suggests the effect of swim was not on retrieval itself, which occurred a day later. The results of Experiment 2b are also consistent with an effect on storage rather than retrieval, because 6 d after the swim, we also observed the enhanced expression of intensively conditioned left/right discrimination memory (Figure 1D). Swim-induced increases in circulating hormones are unlikely to persist for 6 d (corticosterone returns to baseline levels within a day of the forced swim; Figure 1B). This is additional evidence that the effect of the swim was not on the retrieval process itself. Because IHT is conventionally interpreted as indicating memory formation in a “naïve” brain site, perhaps the strongest evidence that the swim altered memory storage and not retrieval is that IHT was induced during the swim (Experiments 7 and 10). An effect on storage rather than retrieval would be consistent with the effects of consolidation, reconsolidation, and protein synthesis inhibition [31], which are all also believed to affect memory storage.

### OCAM, Consolidation, and Reconsolidation

The forced swim activated consolidated memories that were 24 h old, to the best of our knowledge mimicking the basic phenomenon of reconsolidation, possibly with an important distinction. Reconsolidation is said to occur when a consolidated memory is retrieved and the activation converts the memory from a biochemically stable state to a labile state [10],[32] that is characterized by additional memory formation [33] in which the original memory can be modified, strengthened, or changed (see [34] for review). Curiously, we did not observe any memory disruption due to the forced swim stress, which seemed to activate, strengthen, or expand the localization of established memories. Further work is necessary to determine whether the stress-induced activation of memory we observed is biochemically identical to consolidation or reconsolidation, a pair of related but biochemically distinguishable phenomena [35]–[38]. It is important in the present context to point out that both consolidation and reconsolidation are specific to the memory that was directly activated by learning or retrieval [39], whereas we observed that the stressful forced swim activated several different memories, none of which were related to the stressful experience. This distinguishes the stress-induced activation of memory phenomenon we describe from conscious, recall-triggered activation of memory. For example, after CS2 → CS1 → US second-order-conditioning, recall elicited by CS2 causes the directly activated CS2 → CS1 association to become labile without altering the indirectly activated CS1 → US association [39].

We investigated the OCAM effect in several memories, but we only assayed each memory in isolation, so whether stress activates all or a subset of the rat's memories remains an open question. We suspect that the answer will be complex because whether and how a memory is modified after retrieval depends on the strength and age of the memory [40], the brain regions involved in information storage [41], as well as the duration of the reactivation and whether extinction occurred [42]. A model of memory that attempts to synthesize the consolidation and reconsolidation literature [35] states that learning creates a memory trace, and both learning and reactivation evokes memory modulation events. The stabilization of memory is a graded function of the amount of modulation for each memory. This view predicts that forced swim will be more likely to activate recent memories than remote ones. Providing evidence for this hypothesis will require extensive experiments that manipulate both the strength of the memory and the interval between learning and forced swim. However, regardless of whether or not there is a restricted time window during which the stressful swim can cause the modification of a once consolidated memory, the present data demonstrate that OCAM occurs at the very least for consolidated memories that are 24 h old.

### Hippocampus Modulates Extrahippocampal Memories

Blocking hippocampal activity during the swim prevented both the swim-induced memory enhancement and the swim-induced IHT of lateralized memory for left/right discrimination, the learning or expression of which is insensitive to hippocampal inactivation. This suggests that hippocampal activity during the swim was necessary for the out-of-context activation of an extra-hippocampal memory. The results do not indicate whether the role of hippocampus was only to mediate the response to stress or whether hippocampal memories were specifically activated. The data demonstrate the hippocampus plays a role in memory beyond its role in associative memory storage [43]–[45], adding to the evidence that hippocampus modifies recent memories that are stored elsewhere in the brain [8],[46] and is a site along with amygdala for the combined roles of stress and arousal in mediating memory modulation [26],[28].

### Hippocampus-Dependent OCAM

The results of Experiments 8, 9, and 10 suggest that OCAM is a hippocampus-dependent process that appears to alter memory in extrahippocampal sites. OCAM is a common feature of human conscious recollection, but despite a recent suggestion that hippocampus is important for recollection in rats [47], there are alternative interpretations of those data and whether rats recollect remains controversial [48]. To our knowledge OCAM has never been described in non-humans. Future studies will determine whether swim-induced OCAM in rats is related to human out-of-context recollection in part by investigating whether the same hippocampal-neocortical networks are engaged. The electrophysiological re-expression of recently expressed hippocampal and neocortical activity patterns has been recorded from monkey [49] and rat during sleep [50],[51] and during conscious human recall [52]. It is of substantial interest whether such electrophysiological reactivation is the expression of memory and whether it will occur during swim-induced OCAM. Indeed, our extensive use of the intensive aversive L/R discrimination protocol was motivated in part by the fact that it generates stereotyped behavior that is amenable to searching during the stress for replay of the place cell ensemble activity sequences that are expressed during memory formation, as the rat runs up the start arm to the choice point.

### A Hypothesis for Memories in PTSD

The findings presented here indicate that under acute stress, the hippocampus is involved in activating a set of arbitrary memories that can be stored at both hippocampal [41] and extrahippocampal sites. Although we evaluated the effect of stress on single memories, one at a time, we assume that stress can concurrently activate many memories for two reasons. First, the forced swim had little in common with the learning and retrieval experiences we investigated, suggesting stress affects memory in general rather than just memories of specific, stress-related experiences. Second, we observed that the stressful swim enhanced a variety of associations that included a weak appetitively L/R discrimination (Experiment 1A), as well as more persistent aversively conditioned L/R discrimination and inhibitory avoidance responses. These findings extend our understanding of the consequences of memory consolidation and reconsolidation, which our data demonstrate can be modified by stress.

While further investigations of the effects of stress on multiple, concurrent memories are warranted, our observations indicate that stressful experience alters diverse associative memories. We only found evidence of memory enhancement, for both weak and strong associations; it however remains possible that other forms of memory that we did not test were weakened by the stress. Nonetheless, at this point, our observations suggest that in stress-induced OCAM, stress acts to generally strengthen memory rather than acting to strengthen some and weaken others. If confirmed, this may help understand the memory dysfunction in PTSD and other stress-related mood disorders. We hypothesize that stress-triggered memory activation creates a condition where multiple memories coactivate, and through mechanisms of synaptic plasticity [53] that include both long-term potentiation and depression [54]–[57], consolidation and reconsolidation, their subsequent expression is enhanced. We point out that there is evidence that recall which activates a consolidated memory can cause additional information to become incorporated into that memory via the molecular events associated with consolidation [58] but not reconsolidation [59]. According to our hypothesis, already strong traumatic memories or the stress itself can become inappropriately associated with other memories of everyday experience, making the subsequent experience and recall of everyday events more likely to trigger unwanted recall of the traumatic memory.

## Methods

### Ethics Statement

The experiments were conducted in accordance with Institutional (SUNY, IACUC 07-197-05) and NIH guidelines, and the directive of the European Communities Council (6/609/EEC).

### Subjects

Male rats of the Long-Evans strain weighing 350–450 g were used. The experiments were performed during the light period (07:00 to 19:00) of a 12 h:12 h cycle. Rats were habituated to handling by the experimenter for 3–5 d prior to behavioral testing.

### Corticosterone Assay

Trunk blood was collected under Halothane anesthesia. After overnight storage, the blood was centrifuged at 4,000 rpm for 10 min; the supernatant was withdrawn and then stored frozen until assayed by radioimmunoassay.

### Behavioral Procedures

More than 10 experiments were performed requiring the use of a large number of behavioral and experimental manipulations. Here in the Methods we describe the procedures themselves, and to optimize the clarity of the report, we describe each experiment's protocol in an introduction to the individual experiment in the Results.

#### Forced swim

Rats were forced to swim individually for 20 min in a covered bucket (diameter 30 cm) filled to 30 cm with 27°C water. The bucket lid had six small holes to allow air in and the experimenter to observe the rat. Afterwards the rats were dried with paper towels and returned to the home cage. All animals survived the experience and none required additional follow-up care. Unless stated otherwise, the control rats spent the same time in the experimental room and were treated like the experimental animals with the exception that they were not put into the bucket and forced to swim.

#### Appetitive left/right discrimination (Experiment 1)

The rats were food-deprived to 85% of their weight. During 5–6 d they were habituated to the T-maze (45×12×15 cm (l× w × h) arms) and to eat 3 cocoa puffs (General Mills, Minneapolis, MN) during 2 min at the choice point. All rats then received five acquisition trials. A trial began by placing the rat in the start arm and ended when the rat entered a choice arm by half a body length or 120 s elapsed. If the rat entered the goal arm, it was given 3 cocoa puffs. If the rat did not enter a choice arm within 120 s, it was placed in the goal arm, given 3 cocoa puffs, and an error was scored. On Day 3, the rats were allowed 120 s to make a choice on each of three unreinforced retention trials; all rats responded within 120 s.

#### Aversive left/right discrimination (Experiments 2–4, 6–10)

The Y-maze had opaque walls (40×10×30 cm; 120° between arms) and an electrifiable floor made of parallel rods. Each rat was habituated to the maze for 5 min before being trained to escape from a fixed start arm to one of the two choice arms. On each trial, the rat was placed in the start arm and 5 s later foot-shocks (50 Hz, 0.5 mA, 0.5 s) were delivered every 3 s until the rat escaped to the goal arm or 60 s elapsed. The response was correct if the rat escaped directly to the goal, and an error was scored if the rat entered the incorrect arm by at least half of its body. If 60 s elapsed, the rat was put into the goal arm and an error was scored. Each rat was allowed to spend approximately 30 s in the goal arm before it was placed in the home cage (“short” training) or in the start arm (“intensive” training).

#### Short training

In the short training protocol (Experiment 2) the first choice was always considered an error and the other arm was designated the goal. Training continued until either three correct choices or three errors. Rats that made three errors were excluded from the study (*n* = 5). Five retention tests were given on Day 3. The rat was placed in the start arm and shocked after 5 s until it escaped to any choice arm. Immediately afterwards it was returned to the home cage. It was not shocked in any choice arm. The time between trials was about 2 min. The percentage of correct choices was measured.

#### Intensive training

In the intensive training protocol (Experiments 3, 4, and 6–10) the first choice was always considered an error and the other arm was designated the goal. After 9 of 10 consecutive responses were correct, an additional 30 trials were given. Retention was tested by reversal training in which the rats had to escape to the opposite arm than on Day 1 (the Day 1 error arm). The number of errors to the criterion of four consecutive correct responses was used to compare acquisition and retention. More errors during the Day 3 reversal test indicated better retention of Day 1 memory.

#### Inhibitory avoidance (Experiment 5)

The apparatus consisted of two plastic boxes connected with a guillotine door. The brightly lit white start box (30×20×13 cm) had white plastic walls, a metal parallel rod floor, and a Plexiglas ceiling. The dark shock box (25×15×13 cm) had a dark gray plastic ceiling and walls and an electrifiable floor. Rats received two baseline trials and one acquisition trial at 30 min intervals. The rat was placed in the start compartment with its back towards the door, and approximately 5 s later, the guillotine door was raised when the rat was not facing it. After entering the shock compartment, the door was closed and on the baseline trials, 15 s later the rat was returned to its home cage. On the third trial (acquisition), once the rat entered the black compartment, it received two 0.6-mA, 2 s foot-shocks (50 Hz) separated by 1 s, and immediately afterwards the rat was returned to the home cage. Retention was measured without reinforcement by the latency to enter the black compartment. If 300 s elapsed, the rat was removed and the step-through latency was set to 300 s.

### Temporary Functional Lesions

#### Intrahippocampal injection

Rats were implanted under Nembutal anesthesia (50 mg/kg) with a pair of stainless steel injection guide cannulae aimed 1.5 mm above the injection targets in the dorsal hippocampus as described in detail [60]. Training began at least a week after surgery. For intrahippocampal injection, the rat was restrained by hand and a 30-ga injection cannula was inserted into each guide so that the tip was at the target (AP 3.5 mm; lateral 2.6 mm; ventral 3.5 mm). One µl solution (saline; 5 ng TTX/µl saline or 4% lidocaine) was infused during 1 min using a 5 µl Hamilton syringe connected to the cannula by tygon tubing. The cannula was slowly retracted 2 min after the infusion ceased. A habituating injection was given in the home cage a few days before the experiment. Cannula placements were verified to be within 0.5 mm of the target. Figure 5A depicts the injection locations in 20 randomly selected subjects from groups Sw-TTX (Experiment 9, *n* = 10) and CSD-Lid-Sw (Experiment 10, *n* = 10).

#### CSD (Experiments 7 and 10)

The rats were pre-treated with atropine (1 mg/kg) and 5 min later anesthetized by a mixture of ketamine hydrochloride (90 mg/kg) and xylazine hydrochloride (14 mg/kg). Two trephine holes (3 mm diameter) were made over both fronto-parietal cortices without damaging the dura, and each was fitted with an aluminum well (4 mm inner, 6 mm outer diameter, 5 mm high) that allowed free access to the dura in the course of the experiment. This assembly was fixed to the skull with dental acrylic and two anchoring screws. The exposed dura was protected from desiccation by saline-soaked cotton, and both wells were covered with a metal cap. One day was allowed for recovery. Unilateral repeating waves of CSD were elicited using the method of Burešova [61]. A 2 mm ×2 mm piece of filter paper was soaked in 25% KCl and placed on the exposed dura above one hemicortex. In each group, CSD was elicited in the right hemisphere in approximately half the animals and in the left hemisphere in the other rats. After 10 min in the home cage, the CSD was verified by testing for a unilateral impairment of the cortical postural reactions [61],[62]. The same tests were used at the end of each training session. Three animals that did not show a clear absence of the postural and placing reactions on the side opposite to the CSD were excluded from the experiment. After training, the filter paper was removed and the dura was washed with saline and again protected from desiccation. The postural and placing reflexes were observed 60 min after replacing KCl with saline.

### Electroconvulsive Shock (ECS)

Rats were placed in a plastic holding cage next to the forced swim bucket. A pair of electrodes was clipped to the ears and an ECS (50 mA, 50 Hz, 1 s) was delivered. After the treatment, the rats were returned to their home cage to recover.

### Data Analysis

Average measures ± SEM are reported. Significant differences confirmed by ANOVA were followed by Newman-Keuls post hoc tests. The results of these pair-wise comparisons are reported in the main text, and the statistical details are given in the corresponding figure legends. Chi-square and *t* tests were also used as indicated in the text.

## Abbreviations

CSD: cortical spreading depression
ECS: electro-convulsive shock
HPA: hypothalamic-pituitary-adrenal
IHT: inter-hemispheric transfer
OCAM: Out-of-context activation of memory
PTSD: post-traumatic stress disorder
TTX: tetrodotoxin
